# P112 Much Ado About DADA2: two cases of a rare monogenic vasculopathy masquerading as systemic rheumatological disease

**DOI:** 10.1093/rap/rkag113.142

**Published:** 2026-11-03

**Authors:** Eman Elfar, Patrick Kiely

**Affiliations:** St George’s hospital, London, United Kingdom; Somerset NHS trust, Somerset, United Kingdom

## Abstract

**Introduction:**

Deficiency of adenosine deaminase 2 is a rare monogenic autoinflammatory disease that can mimic primary vasculitis, immune deficiency and haematological disease. The key challenge is pattern recognition: identifying when apparently separate diagnoses are manifestations of a single inherited vasculopathy.

**Case description:**

A 44-year-old man was diagnosed aged 28 with microscopic polyangiitis, manifesting with axonal sensory motor neuropathy, pulmonary and ocular vasculitis and abdominal perforation. He had arthritis and myositis (muscle biopsy confirmed). He was treated with intravenous cyclophosphamide, complicated by severe pneumocystis jirovecii and RSV pneumonia. Three years later he had a disabling stroke causing persistent paralysis and wheelchair dependence. He had profound pan-hypogammaglobulinaemia, with IgG 0.9, IgA <0.06 and IgM 0.1 g/L, requiring immunoglobulin replacement. Reassessment found no active synovitis, negative RF/CCP and no active interstitial lung disease, but post infective organising pneumonia and extrapulmonary restriction requiring nocturnal non invasive ventilation. DADA2 was suspected in view of panhypogammaglobulinaemia and confirmed on genetic testing, as a diagnosis unifying his vasculitis, immune deficiency, arthralgia and myalgia. Management shifted from conventional vasculitis maintenance towards Etanercept for secondary stroke prevention and control of systemic inflammatory manifestations.

A 26-year-old man had childhood onset rashes, limb pains, cold sensitivity, abdominal symptoms and low IgA. In 2023, he developed recurrent fatigue, arthralgia, myalgia and a widespread violaceous reticular livedoid rash, with tender axillary nodes, microcytic anaemia, lymphopenia, low IgA/IgM and persistently raised CRP. PET imaging showed intense gastric and terminal ileal uptake. Gastric biopsies revealed gastric marginal zone MALT lymphoma and he received gastric radiotherapy after failed H pylori eradication, with transient improvement then recurring malaise and pain. Rheumatology review identified inflammatory arthralgia without clinical or ultrasound synovitis, livedoid rash on trunk, limbs and hands, CRP 60mg/L, lymphopenia and persistently low IgA/IgM and negative ANA/ENA/Scleroderma/RF/CCP. Repeat PET, axillary node and gastric biopsies and bone marrow aspirate found no evidence of persisting lymphoma. Skin biopsy showed a non-specific myelomonocytic infiltrate. Referral to the national autoinflammatory centre confirmed compound heterozygous ADA2 variants R34W and N370K. He was started on Etanercept.

**Discussion:**

These two contrasting cases illustrate DADA2 as a diagnostic chameleon across paediatric, transitional and adult rheumatology: one presenting after catastrophic vasculitic damage, stroke and severe infection from immune deficiency, the other following childhood onset rash, arthralgia, malaise and raised CRP, followed by gastric MALT lymphoma aged 23 and persisting rash, systemic malaise, arthralgia, lymphopenia, low IgA and IgM and raised CRP.

A mixed phenotype including vascular features (livedoid rash, PAN like vasculitis, stroke, raised CRP), haematologic cytopenias, lymphoma and immune deficiency (hypogammaglobulinaemia, infections) should prompt ADA2 testing. Recognition matters because many features resolve with anti TNF therapy, including suppression of CRP, systemic symptoms and prevention of strokes.

**Key learning points:**

• DADA2 is a rare monogenic autoinflammatory disease with highly variable clinical presentations.

• DADA2 is an important mimic of systemic vasculitis, particularly polyarteritis nodosa.

• The phenotype is heterogeneous, even among patients with the same genetic mutation.

• Clinical suspicion should be raised in patients with early onset, atypical, or treatment refractory vasculitis.

• Recurrent stroke in a young patient should prompt consideration of DADA2.

• Cutaneous manifestations such as livedo racemosa can provide an important diagnostic clue.

• Haematological manifestations, including cytopenias and bone marrow failure, and lymphoproliferative features such as large granular lymphocytes or lymphoma are common.

• Immunodeficiency, including pan-hypogammaglobulinaemia may coexist with inflammatory disease and vasculitis.

• A persistently raised CRP is a feature, but normal inflammatory markers do not exclude the diagnosis.

• Diagnosis should be confirmed with plasma ADA2 enzyme activity and/or biallelic pathogenic variants in the ADA2 gene.

• Early diagnosis is essential to enable treatment to prevent irreversible organ damage and recurrent stroke.

• TNF inhibitors, lifelong, are the first line treatment for the vasculitic and inflammatory manifestations of DADA2.

• Etanercept or Infliximab might be preferred due potential anti-drug antibodies with Adalimumab.

• Corticosteroids are useful for disease control but are not an effective long term treatment.

• Allogeneic haematopoietic stem cell transplantation should be considered in selected patients with severe bone marrow failure.

• Immunoglobulin replacement may be required with prophylactic antibiotics for profound hypogammaglobulinaemia and recurring severe infections

• Management requires close multidisciplinary collaboration between Rheumatology, Immunology, Haematology, Neurology, Genetics, and other specialties.

• Family screening and genetic counselling are important due to the autosomal recessive inheritance pattern.

• Increased awareness across specialties can reduce diagnostic delay and improve patient outcomes.

